# Line-Source Based X-Ray Tomography

**DOI:** 10.1155/2009/534516

**Published:** 2009-04-27

**Authors:** Deepak Bharkhada, Hengyong Yu, Hong Liu, Robert Plemmons, Ge Wang

**Affiliations:** ^1^Biomedical Imaging Division, VT-WFU School of Biomedical Engineering & Sciences, Wake Forest University, Winston-Salem, NC 27157, USA; ^2^Biomedical Engineering Department, Wake Forest University School of Medicine, Winston-Salem, NC 27157, USA; ^3^Biomedical Imaging Division, VT-WFU School of Biomedical Engineering & Sciences, Virginia Tech., Blacksburg, VA 24061, USA; ^4^School of Electrical & Computer Engineering, University of Oklahoma, Norman, OK 73019, USA; ^5^Departments of Mathematics and Computer Science, Wake Forest University, Winston-Salem, NC 27109, USA

## Abstract

Current computed tomography (CT) scanners, including micro-CT scanners, utilize a point x-ray source. As we target higher and higher spatial resolutions, the reduced x-ray focal spot size limits the temporal and contrast resolutions achievable. To overcome this limitation, in this paper we propose to use a line-shaped x-ray source so that many more photons can be generated, given a data acquisition interval. In reference to the simultaneous algebraic reconstruction technique (SART) algorithm for image reconstruction from projection data generated by an x-ray point source, here we develop a generalized SART algorithm for image reconstruction from projection data generated by an x-ray line source. Our numerical simulation results demonstrate the feasibility of our novel line-source based x-ray CT approach and the proposed generalized SART algorithm.

## 1. Introduction

Since the first computed tomography (CT) scanner was made [1], all the commercial scanners have been employing the x-ray source with a small focal spot, which can be mathematically modeled as a point source. In micro-CT and even nano-CT applications, the reduced x-ray focal spot size has become a limiting factor to achieve desirable image resolution in terms of spatial, contrast, and temporal measures. To address this issue, we propose to use a line-shaped x-ray source so that more photons can be generated in a given data acquisition interval. In this context, the x-ray source can be mathematically modeled as a line-segment. In point x-ray source CT scanners, the spatial resolution is limited by the finite focal-spot size necessary to generate a sufficient number of x-ray photons, and the temporal resolution is limited by the time necessary to acquire sufficient projection data over an angular range. 

 In contrast to recently proposed source configurations, like the multiplexed [2] and multiple-source geometry [3] which utilize multiple point x-ray sources to reduce the acquisition time, our technique treats the entire line segment as a single x-ray source. Since a line source covers a wide angular range per view, irradiation with an increased number of photons is achieved along with a relatively higher cooling capability of the x-ray source. Therefore, a line-shaped source technique could be a good candidate to balance among spatial, contrast, and temporal resolution.

A line-shaped x-ray source can be fabricated using field emission x-ray source technology. Field emitters have been used as electron sources for a long time. The most significant difference between the field emission x-ray tube and existing tubes lies in the field emitter cathode. The cathode can be made with an array of micromachined field emission tips. By doing so, it is possible to obtain very sharp tips and very close proximity between the tips and the gate electrode. This greatly reduces the potential difference between the tip and the gate required to achieve the field emission. The array may also be very densely packed. As a result, even though the current that can be obtained from a single tip is small, the total current that can be obtained from an array can be much larger. Schwoebel et al. [4] reported that they were able to obtain a current of 300 mA by packing 50 000 tips into a circular area of a square millimeter, which is equivalent to 40 A/cm^2^. Using the technique as described above, a line-shaped x-ray source can be fabricated. The width can be made as narrow as 0.01 mm (or less), and the length of the source can be made in tens of centimeters or longer, if necessary. 

 The organization of this paper is as follows. In the next section, we formulate a forward imaging model assuming a line source. In the third section, we develop a generalized simultaneous algebraic reconstruction technique (SART) to enable line-source based reconstruction. In the fourth section, we perform numerical tests to demonstrate the performance of our technique. In the last section, we discuss relevant issues and conclude the paper.

## 2. A Line-Shaped X-Ray Imaging Model

**Figure 1:**
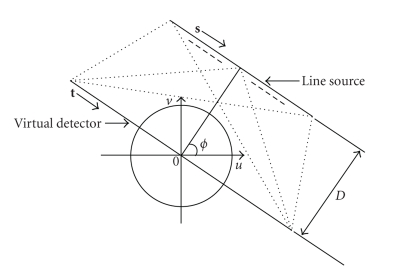
Line x-ray source acquisition geometry.

As shown in Figure 1, a linear virtual detector is assumed in our line-shaped x-ray source acquisition geometry. Vectors **s** = (*u*, *v*) and **t** = (*u*, *v*) refer to the locations of the line-shaped x-ray source and the line detector, respectively. The x-ray forward projection model for a line source in terms of the number of photons arriving at a detector location **t** can be written as

(1) $$N^{O}(t)=\int N^{I}(s)e^{-\int {f\left(x\right)|}_{x\in \text{ray}\left(s,t\right)}dx}ds,$$

where *N* ^*I*^(**s**) is the original number of photons emanating from the source at a point **s**, *N* ^*O*^(**t**) is the number of photons arriving at a location **t** on the detector, **x** = (*u*, *v*) refers to a position along the x-ray path connecting points **s** and **t**, *f*(**x**) is linear attenuation coefficient of point **x**, *x*, and *s*, respectively, represent the 1D coordinates along a fixed ray path and the line source, and the outer integral is carried out along the whole line source. The power of the exponential term (∫* f*(**x**)∣_**x**∈ray(**s**,**t**)_ *dx*) is the line integral of the attenuation coefficients along the x-ray connecting points **s** and **t**.

 If the length of the line is infinitesimally small, the x-ray attenuation model of a point source can be obtained by removing the outer integral in (1) as

(2) $$N^{O}(t)=N^{I}(s)e^{-\int {f(x)|}_{x\in \text{ray}(s,t)}dx},$$

where **s** is the location of the point source and hence can assume only one value per view. Applying the logarithmic transformations on both sides of (2), we can obtain a linear equation representing the line integral of the attenuation coefficients along an x-ray path as

(3) $$p\left(t\right)=-\text{log}\;\left(\frac{N^{O}(t)}{N^{I}(s)}\right)=\int {f(x)|}_{x\in \text{ray}(s,t)}dx.$$

CT image reconstruction is a typical inverse problem of recovering *f*(**x**) from the corresponding forward projection models equations (1) and (3). While there are many analytic algorithms for point-source projection data modeled by (3), the nonlinear nature of (1) for the projection data of a line-shaped x-ray source makes it difficult to obtain a corresponding analytical method for the image reconstruction. However, an iterative method can be developed to achieve the reconstruction as demonstrated in the next section.

## 3. Generalized SART Algorithm

Although iterative methods have not been employed by any commercial CT scanners due to high computational costs associated with them, their superior performance is well established when the data is incomplete, noisy, and dynamic. Meanwhile, there is a renewed interest in iterative algorithms due to the improvement in computational capabilities [5, 6]. It is well known that simultaneous algebraic reconstruction technique (SART) [6, 7] has remained a very powerful tool for iterative reconstruction since its introduction, and it has been shown to converge to a weighted least squares solution from any initial guess [8]. Moreover, the SART method operates in the whole real space, while the other popular iterative algorithm expectation maximization is defined only for nonnegative space, although it does preserve data fidelity for nonnegative pixel and voxel values. Here, we will generalize the SART method for image reconstruction from projection data of the proposed line source model. 

### 3.1. Point Source SART Algorithm

 Because a projection for the point x-ray source is a linear integral of attenuation values along the x-ray path (3), it can be written in a discrete form as

(4) $$p_{i}=\overset{J_{i}}{\underset{j=1}{\sum }}f_{ij}\Delta x,$$

where *p*_*i*_ is the *i*th projection, *i* = 1,…, *I* with *I* being the number of projections given by the product of the number of views and the number of detector pixels, *J*_*i*_ is the number of sample points along the *i*th ray path, *f*_*ij*_ are the attenuation coefficients of the points on the *i*th ray with *j* = 1,…, *J*_*i*_. Because most of the points along the x-ray paths in (4) do not lie on the discrete image grid, it is necessary to obtain the attenuation values *f*_*ij*_ for them from the image grid pixels via interpolation or some other method. Assuming that linear interpolation is employed, we can rewrite (4) as

(5) $$p_{i}=\overset{J}{\underset{j=1}{\sum }}w_{ij}f_{j}\Delta x,$$

where *f*_*j*_ are the attenuation values of the image pixels, *j* = 1,…, *J* with *J* being the number of pixels in the image and *w*_*ij*_ is the weight contribution of *f*_*j*_ on the image grid to the projection *p*_*i*_.

 Let *a*_*ij*_ = *w*_*ij*_Δx. Equation (5) can now be simplified as

(6) $$p_{i}=\overset{J}{\underset{j=1}{\sum }}a_{ij}f_{j}.$$

This is equivalent to a linear system of equations given by

(7) AF=P,

where **A** is a *I* × *J* matrix, **F** is a *J* × 1 vector, and **P** is a *I* × 1 vector with

(8) $$\begin{array}{c} A=\left[\begin{array}{cccccc} a_{11} & a_{12} & \cdots & a_{1j} & \cdots & a_{1J}\\ a_{21} & a_{22} & \cdots & a_{2j} & \cdots & a_{2J}\\ \vdots & & \ddots & & & \vdots \\ a_{i1} & a_{i2} & \cdots & a_{ij} & \cdots & a_{iJ}\\ \vdots & & \ddots & & & \vdots \\ a_{I1} & a_{I2} & \cdots & a_{Ij} & \cdots & a_{IJ} \end{array}\right],\\ \begin{array}{c} F={\left[f_{1},f_{2},\ldots ,f_{j},\ldots ,f_{J}\right]}^{T},\\ P={\left[p_{1},p_{2},\ldots ,p_{i},\ldots ,p_{I}\right]}^{T}. \end{array} \end{array}$$

The SART algorithm for solving **F** from **P** can be written in an iterative format as [6]

(9) $$f_{j}^{\text{iter}+1}=f_{j}^{\text{iter}}+\frac{1}{\sum_{i=1}^{I}a_{ij}(L_{i}/w_{i})}\overset{I}{\underset{i=1}{\sum }}\left(\frac{a_{ij}(p_{i}-a_{i}F^{\text{iter}})}{\sum_{j=1}^{J}a_{ij}}\right),$$

where “iter” indicates the iteration number, **a**_*i*_ is the *i*th row of matrix **A**, *L*_*i*_ is the length of the intersection between the *i*th x-ray path and support of the region being reconstructed and, *w*_*i*_ = ∑_*j* = 1_^*J*^ *w*_*ij*_, and *p*_*i*_ − **a**_*i*_ **F** ^iter^ is the *i*th difference between the real line integral and the line integral estimated from current image **F** ^iter^.

### 3.2. Generalized Line Source SART Algorithm

 Following the same steps as for the point source, a projection for the line-shaped x-ray source based on (1) can be written in the discrete form as

(10) $$N_{k}^{O}=\overset{I}{\underset{i=1}{\sum }}\;N_{i}^{I}\text{exp}(-\overset{J_{ik}}{\underset{j=1}{\sum }}\;f_{kij}\Delta x)\Delta s,$$

where *N*_*K*_^*O*^ is the *k*th projection for the line source, *k* = 1,…, *K* with *K* being the number of projections given by the product of the number of views and the number of detector pixels, *I* is the number of source points chosen for the discretization of the line source, *N*_*i*_^*I*^ is the original number of photons in all x-rays from a line source point *i*, *N*_*ik*_ is the number of sample points along the x-ray path from a line source point *i* to a detector pixel corresponding to the projection *k*, *f*_*kij*_ is the linear attenuation coefficient of the points along the x-ray path from a source point *i* to a detector pixel corresponding to the projection *k*, Δ*x* is the sampling interval along the ray path, and Δ*s* is the sampling interval along the line source. In the following, we assume that all the x-rays from a line source have same original number of photons as *N* ^*I*^. 

 Although (10) is not a linear equation set, we can modify the SART algorithm to solve (10). Because most of the points on the x-ray paths in (10) do not lie on the discrete image grid, again it is necessary to obtain the attenuation values *f*_*kij*_ for them from the image grid pixels. Assuming that linear interpolation is employed, we rewrite (10) as

(11) $$N_{k}^{O}=N^{I}\left(\overset{I}{\underset{i=1}{\sum }}\text{exp}(-\overset{J}{\underset{j=1}{\sum }}w_{kij}f_{j}\Delta x)\right)\Delta s,$$

where *f*_*j*_ are the attenuation values of the image pixels, *J* is the number of pixels in the image and *w*_*kij*_ is the contribution of pixel *f*_*j*_ to the line integral of the attenuation coefficients along the x-ray from a line source point *i* to the detector pixel corresponding to the projection *k*. Let exp(−*d*_*k*_) = *N*_*k*_^*or*^/*N*_*k*_^*oe*^ with *N*_*k*_^*oe*^ being the estimated *k*th projection data and *N*_*k*_^*or*^ the real *k*th projection data, then we have

(12) $$d_{k}=-\text{log}\;\frac{N_{k}^{or}}{N_{k}^{oe}}.$$

The real projection data can now be rewritten in terms of the estimated projection data (11) as

(13) $$N_{k}^{or}=\text{exp}\left(-d_{k}\right)N^{I}\overset{I}{\underset{i=1}{\sum }}\;\text{exp}(-\overset{J}{\underset{j=1}{\sum }}w_{kij}f_{j}\Delta x)\Delta s,$$

which can be simplified as

(14) $$N_{k}^{or}=N^{I}\overset{I}{\underset{i=1}{\sum }}\;\text{exp}(-(\overset{J}{\underset{j=1}{\sum }}w_{kij}f_{j}\Delta x+d_{k}))\Delta s.$$

Again let *a*_*kij*_ = *w*_*kij*_Δ*x*, then (14) can be further simplified as

(15) $$N_{k}^{or}=N^{I}\overset{I}{\underset{i=1}{\sum }}\;\text{exp}(-(\overset{J}{\underset{j=1}{\sum }}a_{kij}f_{j}+d_{k}))\Delta s.$$

Notice that the same *d*_*k*_ is added to the line integrals (∑_*j* = 1_^*J*^ *a*_*kij*_ *f*_*j*_) of the attenuation coefficients along any x-rays from all the line source points to the detector pixel corresponding to the line source projection *k*. 

 Consider a hypothetical situation in which all the points along the line x-ray source serve as point x-ray sources and let *q*_*ki*_ denote the line integrals of the x-rays from these sources contributing to the projection *k*. Although the line integrals *q*_*ik*_ are not known, we will show in the following that it is not necessary to know them. Now we can form a linear system of equations

(16) AF=Q,

where **A** is a *U* × *J* matrix with *U* = *K* × *I*, **F** and **Q** are *J* × 1 and *U* × 1 vectors, respectively, described by

(17) $$\begin{array}{l} A=\left[\begin{array}{cccccc} a_{111} & a_{112} & \cdots & a_{11j} & \cdots & a_{11J}\\ a_{121} & a_{122} & \cdots & a_{12j} & \cdots & a_{12J}\\ \vdots & & & & \ddots & \vdots \\ a_{1i1} & a_{1i2} & \cdots & a_{1ij} & \cdots & a_{1iJ}\\ \;\vdots & & & & \ddots & \vdots \\ a_{1I1} & a_{1I2} & \cdots & a_{1Ij} & \cdots & a_{1IJ}\\ \vdots & & & \vdots & & \vdots \\ \vdots & & & \vdots & & \vdots \\ a_{k11} & a_{k12} & \cdots & a_{k1j} & \cdots & a_{k1J}\\ a_{k21} & a_{k22} & \cdots & a_{k2j} & \cdots & a_{k2J}\\ \vdots & & & & \ddots & \vdots \\ a_{ki1} & a_{ki2} & \cdots & a_{kij} & \cdots & a_{kiJ}\\ \vdots & & & & \ddots & \vdots \\ a_{kI1} & a_{kI2} & \cdots & a_{kIj} & \cdots & a_{kIJ}\\ \vdots & & & \vdots & & \vdots \\ \vdots & & & \vdots & & \vdots \\ a_{K11} & a_{K12} & \cdots & a_{K1j} & \cdots & a_{K1J}\\ a_{K21} & a_{K22} & \cdots & a_{K2j} & \cdots & a_{K2J}\\ \vdots & & & & \ddots & \vdots \\ a_{Ki1} & a_{Ki2} & \cdots & a_{Kij} & \cdots & a_{KiJ}\\ \vdots & & & & \ddots & \vdots \\ a_{KI1} & a_{KI2} & \cdots & a_{KIj} & \cdots & a_{KIJ} \end{array}\right],\\ F={\left[f_{1},f_{2},\ldots ,f_{j},\ldots ,f_{J}\right]}^{T},\\ Q={\left[q_{11},q_{12},\ldots ,q_{1i},\ldots ,q_{1I},|\ldots |,q_{K1},q_{K2},\ldots ,q_{Ki},\ldots ,q_{KI}\right]}^{T}. \end{array}$$

Along the rows of **A** and **Q**, there are two index variables *i* and *k*. If we combine them into one index *u*, we obtain

(18) $$\begin{array}{c} A=[\begin{array}{cccccc} a_{11} & a_{12} & \cdots & a_{1j} & \cdots & a_{1J}\\ a_{21} & a_{22} & \cdots & a_{2j} & \cdots & a_{2J}\\ \vdots & & \ddots & & & \vdots \\ a_{u1} & a_{u2} & \cdots & a_{uj} & \cdots & a_{uJ}\\ \vdots & & \ddots & & & \vdots \\ a_{U1} & a_{U2} & \cdots & a_{Uj} & \cdots & a_{UJ} \end{array}],\\ Q={\left[q_{1},q_{2},\ldots ,q_{u},\ldots ,q_{U}\right]}^{T}. \end{array}$$

At first look it appears that we can now use (9) only if **Q** is known as

(19) $$f_{j}^{\text{iter}+1}=f_{j}^{\text{iter}}+\frac{1}{\sum_{u=1}^{U}a_{uj}(L_{u}/w_{u})}\overset{U}{\underset{u=1}{\sum }}\left(\frac{a_{uj}(q_{u}-a_{u}F^{\text{iter}})}{\sum_{j=1}^{J}a_{uj}}\right),$$

where *L*_*u*_ is the length of the intersection between the *u*th x-ray path and support of the region being reconstructed and *w*_*u*_ = ∑_*j* = 1_^*J*^ *w*_*uj*_. Notice that *q*_*u*_ − **a**_*i*_ **F** ^iter^ is the difference between the real line integral *q*_*u*_ and the line integral **a**_*i*_ **F** ^iter^ estimated from the current iterate value **F** ^iter^ of the image. This difference for a particular projection *k* is given by *d*_*k*_ obtained in (12) thus making it unnecessary to know *q*_*u*_. 

 To be consistent with indexing, let us also call *d*_*k*_ as *d*_*u*_ while remembering that *u* is a combination of indexes *i* and *k* and that *d*_*u*_ is the same for all the x-rays (all *i*'s) from the line source for a particular projection *k*. We can now write (19) as

(20) $$f_{j}^{\text{iter}+1}=f_{j}^{\text{iter}}+\frac{1}{\sum_{u=1}^{U}a_{uj}(L_{u}/w_{u})}\overset{U}{\underset{u=1}{\sum }}\frac{a_{uj}d_{u}}{\sum_{j=1}^{J}a_{uj}}.$$

Equation (20) is the final SART algorithm for image reconstruction from the projection data of a line-shaped x-ray source. Because it can be applied for image reconstruction from any nonlinear projection model, we call it a generalized SART algorithm.

## 4. Numerical Simulations

To validate the feasibility of line-shaped x-ray source and demonstrate the merits of generalized SART algorithm, we developed a numerical simulator. The thorax Phantom [9] was used in our simulations. A 60 cm virtual linear detector was assumed, and its detector element size was 0.1 cm. The center of the line source was at a perpendicular distance (*D*) (see Figure 1) of 75 cm from the iso-center. The whole line source was rotated with the center of the line source tracing a circle of radius 75 cm. The source lengths of 3 cm, 5 cm, and 8 cm were employed with projection data acquired for 160 equiangular views. The number of photons used per x-ray per source point was assumed to be 10^7^.

**Figure 2:**
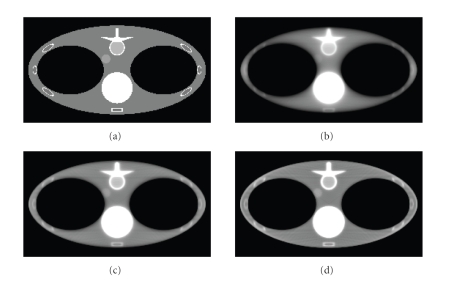
Comparison of images reconstructed using line-SART algorithm using a line source of length 3 cm at different iteration numbers. (a) Original Image. Reconstructed images in (a), (b), and (c) are, respectively, at 30, 60, and 100 iterations. Display window is [0.8, 1.2].

**Figure 3:**
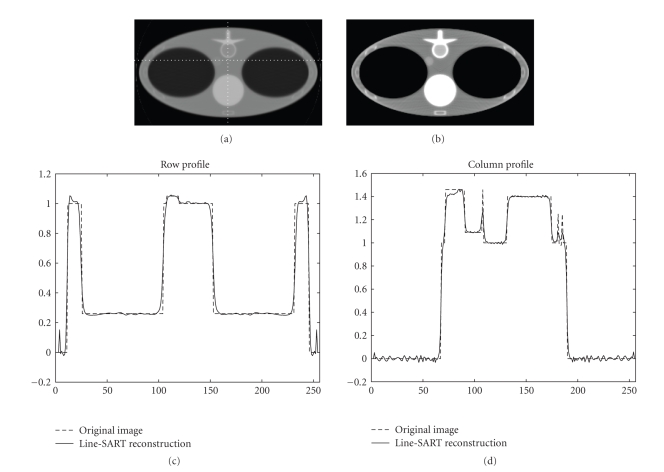
Line SART reconstructed images after 100 iterations using a line source of length 3 cm at two different display windows with profiles along the dotted line. (a) Reconstructed image at a display window of [0, 2]. (b) Reconstructed image at a display window of [0.8, 1.2]. (c) Profile of the row. (d) Profile of the column.

**Figure 4:**
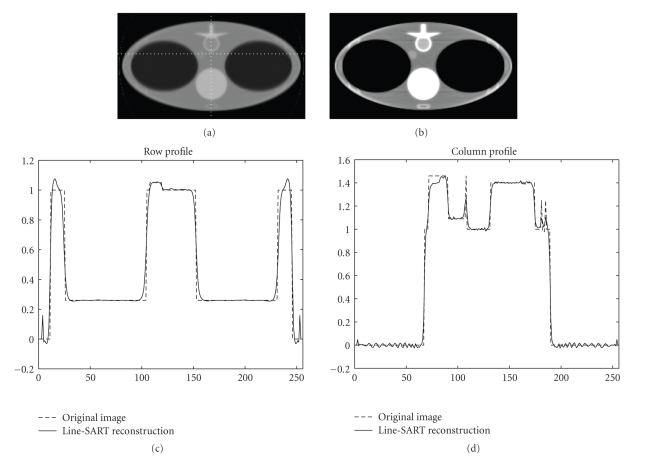
Line SART reconstructed images after 100 iterations using a line source of length 5 cm at two different display windows with profiles along the dotted line. (a) Reconstructed image at a display window of [0, 2]. (b) Reconstructed image at a display window of [0.8, 1.2]. (c) Profile of the row. (d) Profile of the column.

**Figure 5:**
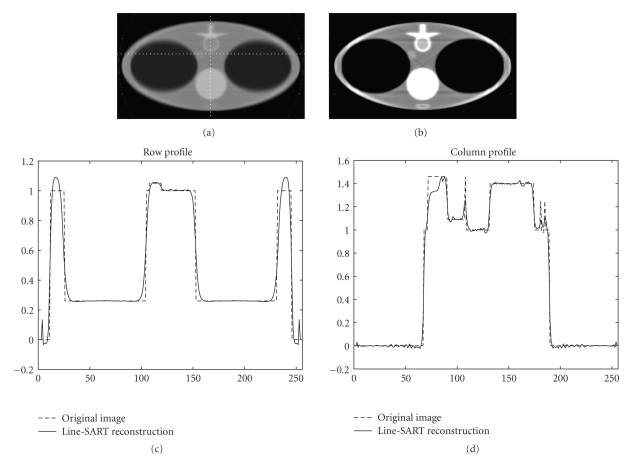
Line SART reconstructed images after 100 iterations using a line source of length 8 cm at two different display windows with profiles along the dotted line. (a) Reconstructed image at a display window of [0, 2]. (b) Reconstructed image at a display window of [0.8, 1.2]. (c) Profile of the row. (d) Profile of the column.

Numerical simulation results are presented in Figures 2, 3, 4, and 5. The size of the images presented is 23.625 × 42 cm^2^ with 144 × 256 pixels. In Figure 2, images are reconstructed at different number of iterations for a line source length of 3 cm. Comparing Figures 2(b), 2(c), and 2(d), it can be observed that increasing number of iterations results in images with sharper edges. This phenomenon is more obvious at the edges of the vertebra and the lungs. Image reconstructed after 100 iterations for the x-ray source lengths of 3 cm, 5 cm, and 8 cm along with the column and the row profiles are presented in Figures 3, 4, and 5, respectively. For our simulation cases the best image quality is obtained with a source length of 3 cm, and relatively poor image quality is obtained with the source lengths of 3 cm and 8 cm. The increase in source length results in increased blurring especially at the edges. Thus, it becomes increasingly difficult to reconstruct fine structures which in our case are the ribs since they have sharp and thin boundaries.

## 5. Discussions and Conclusions

 A point x-ray source used in commercial CT scanners limits the temporal and spatial resolution and also results in frequent heating of the x-ray tube. These limitations may be overcome by using the proposed line-shaped x-ray source based image technique. Reconstructing image from a line-shaped x-ray source is a challenging task due to nonlinear nature of the resulting projection data. In this article, we developed a generalized SART algorithm to enable reconstruction from a line source. We believe that this algorithm can be easily extended to more general 1D x-ray source shapes and even to 2D planar x-ray sources as well, which may have applications in dynamic imaging. Moreover, the OS-SART algorithms [10] can be similarly modified to obtain a generalized OS-SART algorithm with faster convergence.

 To the best of our knowledge, this is the first paper attempting to solve the contradiction between temporal and spatial resolutions by a nonpoint source. Additional research efforts are necessary along this direction. In this study we acquired projection data over an angular range of 2*π*, but it will be interesting to find out for practical reasons the minimum angular range necessary for reconstruction which may change for different source lengths. It will also be exciting to see if a finer sampling of x-ray source during numerical simulations improves spatial resolution. Although it has not been theoretically proved, we believe that the generalized SART is convergent to the real image value, as shown for SART in [8]. However, a detail study is beyond the scope of this paper but will provide additional insights into the performance of the algorithm. 

 There are also possibilities for enhancing our algorithm or developing new algorithms. In our view, the main limitation of our generalized SART algorithm is the blurring of the edges and a large number of iterations required to reconstruct sharp images. To this end, gradient or prior information may be incorporated within our algorithm or other algorithms utilizing gradient information could be developed in the future. Some examples of prior information include support and nonnegativity constraints [11]. In addition, investigation of regularization approaches to alleviate noise magnification and blurring artifacts [12] is an important research direction to pursue in future work. 

 This is a feasibility study, and there is not enough information to comment on scatter and its effects. Scattering is a real concern in computed tomography and it will become important to study the amount of scatter and resulting image degradation as the line-shaped-based x-ray source tomography advances. However, if needed, new algorithms could be developed to reduce the effects of scatter.

 In conclusion, we proposed a novel line-shaped x-ray source based CT imaging technique and corresponding reconstruction method. The developed generalized SART algorithm enables image reconstruction from projection data of not only a line-shaped x-ray source but also more generalized 1D and 2D source. Our numerical simulations have demonstrated the feasibility and merits of the proposed techniques and algorithms. Some interesting future research directions were also presented.
